# Does anti-inflammatory drugs modify the severe odontogenic infection prognosis? A 10-year’s experience

**DOI:** 10.4317/medoral.23926

**Published:** 2020-10-09

**Authors:** Candice Delbet-Dupas, Laurent Devoize, Aurélien Mulliez, Isabelle Barthélémy, Nathalie Pham Dang

**Affiliations:** 1 MD. Hospital Practitioner, Department of Oral and Maxillofacial surgery, NHE – CHU de Clermont-Ferrand, Université d’Auvergne, France; 2 DDS, PhD, Professor. Head of the Department of Odontology, CHU de Clermont-Ferrand, Université d’Auvergne, France; 3 Academic teacher's/researcher’s of UMR Inserm/UdA, U1107, Neuro-Dol, Trigeminal Pain and Migraine, Université d’Auvergne, France; 4 PhD, Biostatisticien. Délégation Recherche Clinique and Innovation, CHU de Clermont-Ferrand, Université d’Auvergne, France; 5 MD, PhD, Professor. Head of Department of Oral and Maxillofacial surgery, NHE – CHU de Clermont-Ferrand, Université d’Auvergne, France; 6 MD, PhD, Hospital Practitioner. Department of Oral and Maxillofacial surgery, NHE – CHU de Clermont-Ferrand, Université d’Auvergne, France

## Abstract

**Background:**

Numerous biochemical datas support the noxious role of anti-inflammatory drugs on immune response. Those observations are often put forward for unfavorable evolution of odontogenic infection but has never been really proven in clinic. The aim of this study is to try to clarify this role based on the collection of the clinical course of odontogenic infections over a 10-year analysis period.

**Material and Methods:**

The investigators implemented a prospective observational study. The sample was composed of patients managed between January 2004 and December 2014 for severe odontogenic infection based on three criteria: hospital admission, intravenous antibiotic therapy, tooth extraction and collections drainage under general anesthesia. Clinical and pharmacological data were collected at admission, during hospitalization until discharged home. The population was first separated into two groups patients with or without anti-inflammatory drugs on admission, then on four groups (non-steroidal anti-inflammatory drugs, corticosteroids drugs, both and none on admission). Analysis were performed each time by univariate analysis, multivariate analysis and propensity score matching.

**Results:**

Six hundred and fifty-three patients were included in the study, 329 (50%) patients report orally anti-inflammatory treatment before presenting to hospital, 50 (7.6%) received corticosteroids, 242 (37%) received NSAIDs and 37 (5.6%) both. Evolution is worsening for patients under anti-inflammatory drugs in term of hospitalization in ICU (*p*=0.016), number of surgeries (*p*=0.003), risk of tracheotomy (*p*=0.036), duration of hospitalization (*p*=0.005) and spaces involved by the infection (*p*<0.001). When separating patients into 4 groups, dysphonia and odynophagia are more frequent for patients under corticosteroid and NSAID (35.14%, *p*<0.001), mediastinal erythema is more frequent for patients under corticosteroid (16%, *p*=0.004), fever is more frequent for patients under NSAID (35.5%, *p*=0.032), pain is higher for patients under corticosteroids (*p*=0.024). But, in order to reduce bias, linked to factors of gravity, a regression weighted by propensity scores was performed and any group of patients is different from the others.

**Conclusions:**

Patients under anti-inflammatory drugs have more severe dental infection on admission and their complex evolution seems to be linked to the severity of infection on admission.

** Key words:**Severe odontogenic infection, anti-inflammatory drugs, corticosteroids.

## Introduction

Dental infection is a common pathology. Surgical incision and drainage of the purulent collections in combination with concerned tooth extraction or treatment, oral cavity rehabilitation and probabilistic antibiotic therapy remain the principles of the treatment (1). Nevertheless for over ten years, acute dental infection management is not so longer simple: more and more patients need long-time hospitalization, several surgical interventions and intensive care follow-up to heal (2-5) . Moreover, in a unclear way, patients with few clinical symptoms of severity (as dysphonia, dyspnea, anterior floor edema, limitation of tongue protraction, oropharyngeal edema) on admission may have unfavorable evolution (2,4,6).

One of the biggest patient’s complaint is pain. To alleviate pain, paracetamol in association with tramadol or codeine is often insufficient whereas the addition of anti-inflammatory drugs quickly improves the discomfort. This practice remains controversial. Yet, the mechanism of action of anti-inflammatory drugs includes an inhibitory effect of immune defenses. Non-steroidal anti-inflammatory drugs (NSAID) may inhibit neutrophil functions, either aggregation or degranulation, both *in vitro* and *in vivo* (7) and may inhibit prostaglandin synthesis (8). This effect may consequently promote the onset or the aggravation of infectious processes normally controlled by an immune physiological response. This potentially noxious role is often mentioned for glucocorticosteroids, but also mentioned for NSAID (9). NSAID may also promote bacterial multiplication (10). In the literature, implication of glucocorticoids and NSAID therapy in initiation or aggravation of cervico-facial cellulitis of dental origin is unclear (9,11-15). To date, a causal link between cervico-facial cellulitis and glucocorticoids and/or NSAID has not been established. Empirically, in our practice, we suspect that patients under glucocorticoids and/or NSAID therapy have a high frequency of complications, of diagnosis delay and stay more often in ICU. Our clinical observation initiated this observational study to determine if glucocorticoids and/or NSAID therapy increase severity and complications of dental infection.

## Material and Methods

- Recruitment

This prospective observational study was conducted between January 2004 and December 2014 in the Department of Oral and Maxillofacial Surgery. In accordance with the ethical principles of the World Medical Association Declaration of Helsinki for medical research involving human subjects (CE-CIC-GREN-12-08).

Adult patients who met the following criteria: acute dental infection which need hospitalization in the department of Oral and Maxillofacial surgery, Clermont-Ferrand University Hospital (France), intravenous antibiotics and surgical management with tooth extraction, incision and drainage under general anaesthesia were enrolled. Non-inclusion criteria were pregnancy, limitations of self-expression, and patients under tutorship or curatorship.

- Data collection

The following data were collected:

1) socio-demographic data: age, sex, associate medical condition as penicillin allergy, smoking status, consumption of alcohol, psychiatric disorder, immunodepression (long-term diabetes, obesity, asthma, chemotherapy, HIV and hepatitis B or C infection, transplant surgery);

2) number and type of space involved by the infection and tooth concerned;

3) treatment on admission: ongoing antibiotic and/or anti-inflammatory treatment;

4) duration of hospitalization and number of surgeries;

5) bacteriological results, results of culture and sensitivity testing;

6) antibiotics adaptation;

7) complications (16)

- Patient management

Severe odontogenic infections are managed with a standardized protocol. Medical workup was initially performed, including history, clinical examination, and C - Reactive Protein (CRP) assay and anesthesiologist consultation. For all patients, NSAID and corticotherapy prescribed in the context of dental infection was stopped on admission in our Department. Preoperative images were dental panoramic and cervicofacial CT-scan in case of clinical symptoms of severity. In the operative room, airways were secured and patient anesthetized. Aseptic conditions required for any surgery were respected, Povidone betadine (ASTA Medica) was used as antiseptic solution to disinfect the facial, cervical area and oral cavity, Chlorhexidine 2% was used in case of allergy to iodine-based products. Causal tooth was extracted to liberate purulent flow. At this time, sampling of pus was performed. Bacteriological samples are cultured in aerobic and anaerobic conditions. Incision, drainage and debridement were performed for all anatomic cervical fascial spaces involved by infection. Collections were drained with intraoral or transcervical or combined approach. Delbet drains were placed through the opened incisions and retained with a suture to realize large lavages by 0.9% saline. Every day, patients had clinical examination, irrigation and dressing. Postoperative CT-scan, and CRP assay were based on patient’s progress. After surgery, all patients were fed by naso-gastric tube to enable drainage and oral healing.

All patients received intravenous probabilistic antibiotherapy effective against oral mucosa flora. The French Health Products Safety Agency (17) (ANSM) recommendations are monoantibiotherapy by Amoxicillin and Clavulanic Acid (1 gramme every 8 hour); in case of penicillin allergy: Clindamycin (300 mg every 8 hours) and Metronidazole (500 mg every 8 hours). Secondly, if necessary, antibiotics were adapted to bacteriological results. (Recommendations by the French Health Products Safety Agency, 2003).

- Statistical analysis

Statistical analysis was performed using Stata V15 (StataCorp, College Station, Texas, USA). All tests were two-sided and a *p-value* <5% was considered statistically significant. Study sample is described by frequency and percentage for categorical data and using means±standard deviation (or median and interquartile range when data not normal) for continuous data. Treatment groups (NSAID or CS vs no NSAID nor CS) and binary outcomes (ICU hospitalization, more than 1 surgery, Hospitalization over 10 days) were compared using chisquared test (or Fisher's exact test when appropriate) for categorical data and using Student's t test (or Mann & Whitney's test when data not normal) for continuous data. Four treatment groups (NSAID, CS, NSAID+CS and no NSAID nor CS) were compared using chisquared test (or Fisher's exact test when appropriate) for categorical data, and using analysis of variance (or Kruskal-wallis test when data not normal) for continuous data. A multivariate logistic regression model was performed to analyze the outcomes, adjusted for factors that were clinically relevant or statistically significant I univariate analysis. Results are show as adjusted odd ratios and their 95% confidence interval. In order to take into account for possible confounding factors in the relationship between outcomes and treatments, we identified those factors (the ones which were significantly both related to outcomes and treatments), and we performed a propensity score using multivariate logistic regression with treatment as dependent variable and with the confounding factors as independent. This model lead to a propensity score for each patient. Then we performed the analyses of the relationships between outcomes and treatments using inverse propensity score weighting, using chisquared test in univariate analysis and a multivariate logistic regression model (weighted on inverse propensity score).

## Results

- Baseline characteristics

A total of 653 patients were recorded, 386 (59%) male and 267 (41%) female with a mean age of 37 years (range 8 – 88). Of them, 375 (57%) were smokers, 78 (12%) regular drinkers, 54 (8%) addicts to drugs, 62 (9.5%) had psychiatric disorders and 20 (3%) were immunodepressed. Forty-seven patients (7.2%) were allergic to penicillin.

Among all patients, 378 (58%) had been receiving oral antibiotics before going to hospital. The treatment was prescribed by their general practitioner, dentist or emergency doctor. There were also a few cases of self-medication. Treatment duration before presentation at the Oral and Maxillofacial Department was 4.1 days (range 1 – 30). Most patients (231; 61%) were prescribed amoxicillin or amoxicillin and clavulanic acid; 75 (20%) had a combination of spiramycin and metronidazole, 37 (10%) pristinamycin, 36 (9.5%) metronidazole and 2 (0.5%) clindamycin.

A total of 329 (50%) patients had orally anti-inflammatory treatment before presenting to hospital, 50 (7.6%) received corticosteroids, 242 (37%) received NSAIDs and 37 (5.6%) both, 261 (40%) received no anti-inflammatory drugs and for 63 (10%) the information about this treatment is unknown or unclear. A total of 238 (36%) had received an association of both orally anti-inflammatory ant antibiotic treatment. On admission, NSAID was stopped for all concerned patients and corticosteroid drugs were stopped only for patients for whom the molecule was prescribed because of the odontogenic infection.

Among 645 patients with collected data, 516 patients (80%) presented during surgery a single facial space involvement by pus, 95 patients (15%) had 2 spaces, 28 (4%) had 3 spaces, 5 patients (1%) had 4 spaces and 1 patient had 6 spaces involved. The submandibular space and the vestibular space were the most frequently involved, respectively 32% (n=209) and 28% (n=182).

Among the 653 patients, 611 (94 %) had no further surgery. Forty-two patients (6%) had more than one surgery (range 2 – 15 surgeries); their mean hospitalization duration was 14.6 days (range 5 – 37 days). In this group, 2 patients had mediastinitis, 5 had cervical necrotizing fasciitis, 21 were hospitalized in an intensive care unit, 13 needed a tracheotomy and 1 patient died of acute respiratory distress associated with sepsis.

- Comparison of the two patient groups anti-inflammatory (NSAID and/or corticosteroids) drugs prescribed before admission versus no NSAID and no corticosteroid treatment (Table 1)

We compared two groups of patients: group 1+2+3 (patients under NSAID or corticosteroids on admission; n = 329 50.4%) and group 4 (patient under no anti-inflammatory treatment; n = 261; 40%); for 63 (10%) patients’ information is unknown or unclear about treatment status on admission.

**Table 1:**
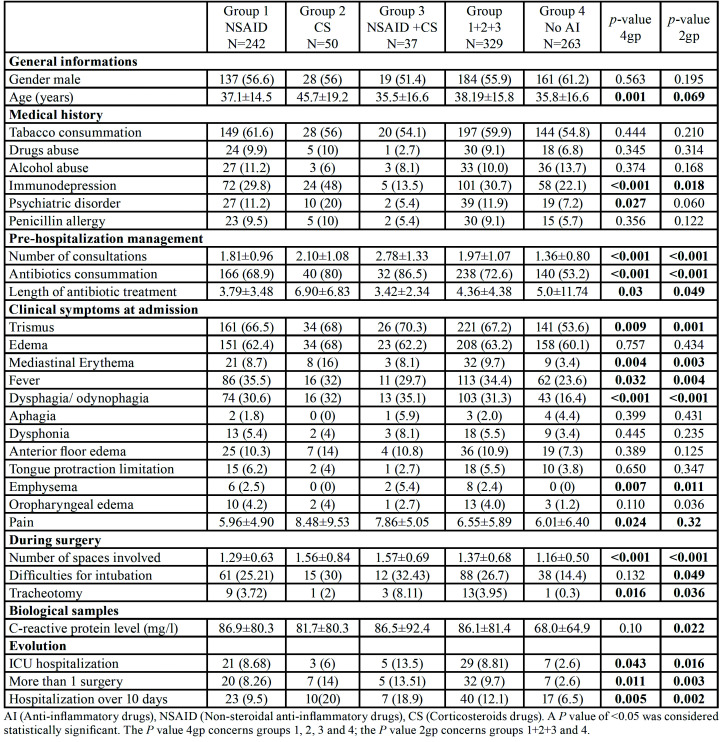
Univariate analysis comparing patients not under anti-inflammatory drugs (Group 4, n=263), patients under NSAID (Group 1, n=242), patients under corticosteroids (Group 2, n=50) with patient both NSAID and corticosteroids (Group 3, n=37). Univariate analysis comparing patients under anti-inflammatory drugs (Group 4, n=263) with patients not under anti-inflammatory drugs (Group 1+2+3, n=329).

The two groups were homogenous in term of gender, tobacco consummation, alcohol and drug abuse and tooth involved; however in group 1+2+3 patients, were older (35.8 years in group 4 and 38.2 years in group 1+2+3, *p*= 0.069) and more frequently immunodepressed (22.1% in group 4 and 30.7% in group 1+2+3, *p*< 0.001). Before admission, patients from group 1+2+3 have more consulted (1.97+/-1.07 versus 1.36+/- 0.80, *p*< 0.001) and were more frequently under antibiotic treatment (72.6% versus 53.2% (*p*= 0.000)). The signs of gravity on admission are more frequent in group 1+2+3: trismus (*p*=0.001), mediastinal erythema (*p*=0.003), fever (*p*=0.004), dysphagia and odynophagia (*p*<0.001), emphysema (*p*=0.011) and oropharyngeal edema (*p*=0.036). Concerning the other symptoms of gravity as edema, aphagia, dysphonia, anterior floor edema and tongue protraction limitation, no statistically difference was observed. Evolution is worsen for patients of group 1+2+3; hospitalization was more frequent in ICU (*p*=0.016); they needed more than one surgery to drain all collections (*p*=0.003); intubation was more often considered as difficult (*p*=0.049); tracheotomy was more frequently performed (*p*=0.036); hospitalization was more often longer than 10 days (*p*=0.002) and more spaces were involved by the infection (*p*=0.003). The univariate analysis reveals a significant difference between the two groups (any anti-inflammatory and with anti-inflammatory treatment) ongoing. Patient who had consuming anti-inflammatory are more to need several interventions, longer hospitalization duration, hospitalization in ICU and tracheotomy. At this level of lecture, anti-inflammatory treatment was obviously linked with more severe odontogenic infection and adverse evolution.

- Comparison of the four patient groups, NSAID, corticosteroid, NSAID with corticosteroid prescribed before admission and no NSAID nor corticosteroid on admission (Table 1)

Because NSAID and corticosteroids have different mechanism of action against inflammation process we decided to share our cohort of patients in 4 groups according to the anti-inflammatory drug prescribed to the patient: group 1 (patients under NSAID on admission; n = 242, 37%), group 2 (patients under corticosteroids on admission; n = 50, 7.6%), group 3 (patients under both NSAID and corticosteroid on admission; n = 37, 5.6%) and group 4 (patient under no anti-inflammatory treatment; n = 263, 40.3%).

Those four groups were also homogeneous in terms of gender, tobacco consummation, alcohol and drug abuse but patients under corticosteroids are significantly older (*p*=0.001) and immunosuppressed patients have a higher probability to be treated by corticosteroids before admission (*p*<0.001).

There was significant difference concerning the pre-hospitalization management. Patients under corticosteroids alone or in association with NSAIDS have had a higher number of consultations before being hospitalized (*p*<0.001) and are more frequently under antibiotic therapy (*p*<0.001). Length of antibiotic therapy is higher in group 2 (6.90 days; SD=6.83; *p*=0.03).

Dysphonia and odynophagia are more frequent for patients under association of NSAID and corticosteroid (35.1%, *p*<0.001) as trismus (70.3%, *p*<0.009). Mediastinal erythema is more frequent for patients under corticosteroid alone (16%, *p*=0.004) as pain with a level of 8.48 in the Numeric Rating Scale (*p*=0.024). Fever is more frequent for patients under NSAID (35.5%, *p*=0.032). Clinical symptoms of gravity as dysphagia and odynophagia, trismus, mediastinal erythema, emphysema, fever and pain are significantly different between groups and systematically higher in groups 1, 2 and 3 in relation to group 4. There was no significant difference between groups and tooth involved.

Concerning the anesthesiologic and surgical management, patients under corticosteroid and NSAID have a higher risk to need tracheotomy (*p*= 0.016) and more spaces involved by infection (*p*<0.001). Patients under corticosteroid in association with NSAID stay more frequently in ICU (*p*=0.043) and have a higher risk to need several surgeries (*p*=0.011). Patient under corticosteroids alone are more frequent to stay longer in hospitalization (*p*=0.005).

Patients under corticosteroids alone or in association with NSAID have consulted more practitioners before admission and have had more antibiotics prescribed. They have also more symptoms of gravity as mediastinal erythema, pain, dysphagia and odynophagia, and more space involved. Evolution is much more complex with larger number of patients who need to stay in ICU, to return to operative room and to need a tracheotomy. At this level of interpretation, corticosteroid treatment seems to be linked with more severe odontogenic infection and adverse evolution.

- Comparison of the four patient groups, NSAID, corticosteroid, NSAID with corticosteroid prescribed before admission and no NSAID nor corticosteroid on admission using propensity scores and multivariate analysis (Table 2, Table 3)

In order to reduce bias, linked to factors of gravity, that may by definition increase the risk of complications, An analysis using inverse propensity score weighting was performed. The confounding factors identified by the statistician and the clinician were statistically significant and also linked to the group of patients under inflammatory-drugs and to the outcome. Those factors were immunodepression, number of spaces involved, fever, dysphagia and odynophagia, mediastinal erythema and emphysema.

**Table 2:**
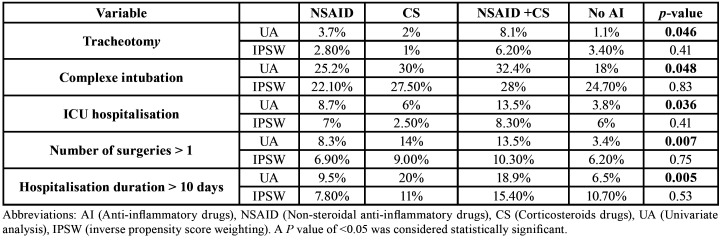
Univariate analysis and inverse propensity score weighting comparing patients under NSAID, patients under corticosteroids, patients both under NSAID and corticosteroids with patients not under anti-inflammatory drugs concerning the risk to have a complex evolution. When performing a univariate analysis, patients under NSAID drugs or corticosteroids drugs or both of them have a higher risk to have a tracheotomy, a complex intubation, a hospitalization in ICU, more than one surgery or a hospitalization duration longer than 10 days. After applying the propensity score weighting regression no statistical difference is emphasis between the 4 groups.

**Table 3:**
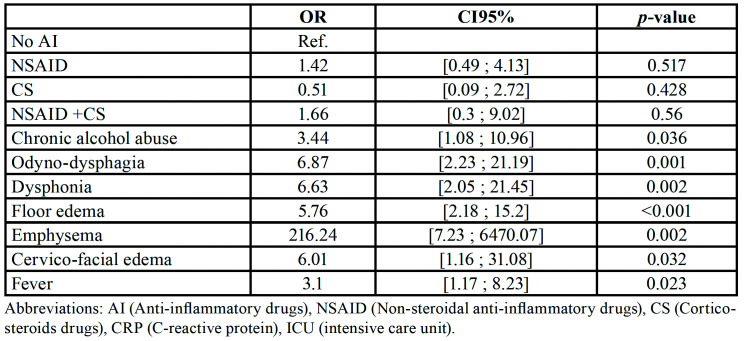
Multivariate analysis concerning the risk to stay in ICU. In the multivariate analysis, gravity criteria as chronic alcohol abuse, odyno-dysphagia, dysphonia, floor edema, emphysema, CRP level, cervico-facial edema and fever are the only variable linked with a higher risk to be hospitalized in ICU; NSAID drugs or corticosteroids drugs or both of them consummation are not linked to a higher risk to stay in ICU.

When applying the inverse propensity score weighting on the number of surgeries performed before healing, 6.9% of patients treated by NSAID had more than 1 surgery, 9% of patients treated by corticosteroids, 10.3% of patients treated by both NSAID and corticosteroids and 6.2% of patients not treated by anti-inflammatory drugs (*p*=0.75). Concerning the hospitalization duration, 7.8% of patients treated by NSAID staid more than 10 days, 11% of patients treated by corticosteroids, 15.4% of patients treated by both NSAID and corticosteroids and 10.7% of patients not treated by anti-inflammatory drugs (*p*=0.53). Concerning the percentage of complex intubation, tracheotomy and ICU hospitalization after applying the propensity score weighting regression no statistical difference is emphasis between the 4 groups (Table 2). To complete the data, a multivariate analysis was performed to determine the risks factors to stay in ICU (Table 3). Any group of patients is different from the others concerning anti-inflammatory drugs consumption.

## Discussion

In most cases of severe odontogenic infection, the cellulo-adipose spaces of the head and neck are involved by pus but tooth extraction, drainage of the collections and systemic probabilistic antibiotic therapy rapidly resolves the infection (18). Yet, in some rare but unclear cases, life-threatening is engaged due to an extensive necrosis and a rapid spreading to mediastinum (2,4,19). This prospective observational study showed that patients managed in our Department of Oral and Maxillofacial surgery for acute dental infection have significantly more complex evolution especially when taken anti-inflammatory drugs. But they also have significantly more symptoms of gravity as mediastinal erythema, fever, pain, dysphagia and odynophagia, and more spaces involved. Patients under anti-inflammatory drugs have more severe acute dental infection and their complex evolution seems to be linked to the severity of infection before admission.

Two explanations could be found: 1/ the use of anti-inflammatory drugs might be related to an immediately aggressive infectious process where oral antibiotic therapy did not success with corollary increased inflammation and pain. 2/ or anti-inflammatory drugs are suspected to impact directly the evolution to severe cervico-facial infections through lower immune response. A biochemical rationale support the second theory, indeed they enhance production of tumor necrosis factor (a key mediator of septic shock) and tissue levels of interleukin 6; also suppress neutrophil functions, which leads to attenuate the manifestations of inflammation (14,20,21). Anti-inflammatory drugs may mask the signs and symptoms of infection, delaying diagnosis and treatment.

In 1985, Brun *et al*., reported a correlation between NSAID use and necrotizing fasciitis involving group Aβ - hemolytic streptococcus (Streptococcus pyogenes) occurring in otherwise healthy patients (11). Two experimental studies demonstrated that group A streptococcal soft tissue infection is worsened by NSAIDs. Weng *et al*., reported that ibuprofen –treated mice exhibited more evident macrophage infiltration and tissue damage in Group A streptococcus infected soft tissue and a death rate of 72.5% whereas all mice without ibuprofen therapy survived (14). Hamilton *et al*., on rats, showed that all nonselective NSAIDs significantly accelerated mortality and reduced antibiotic efficacy (22). By contrast, a rat model, mimicking a cervical necrotizing fasciitis treated either by diclofenac, either by saline, did not supported the hypothesis that NSAID increase the severity of infection (13). In parallel with the reported works, case-series reported that patients with severe odontogenic infection under NSAID therapy have a higher risk to present a mediastinitis or to be admitted in an intensive care unit (9,15). However, Aronov *et al*., review reported that most retrospective case series or case-control studies do not support a relation between initiation or aggravation of necrotizing cellulitis and NSAID use, moreover results from prospective studies suggest that the incidence and the severity of necrotizing fasciitis are not increased in patient receiving NSAID therapy (12). No studies report a certain relation between severe soft tissue infection and corticosteroids. Actually, the potential risk of worsening odontogenic infection to a cervicofacial extensive cellulitis associated through use of anti-inflammatory drugs is still questioned.

The numerous clinical and epidemiologic studies did not resolved this hypothesis (21). Our study has some limits, it was not possible to distinguish patients with long-term corticosteroids for general disease and patients under corticosteroids for the odontogenic infection. We could not collect the NSAIDs or corticosteroids dose absorbed by the patients to research a dose-dependent product activity.

This work did not allow demonstrating the action of anti-inflammatory drugs in worsening severe odontogenic infection but it demonstrate that patient under anti-inflammatory drugs have more severe acute dental infection on admission with de facto a higher risk of complications. This support the hypothesis that anti-inflammatory drugs may mask the symptoms of infection and delay diagnosis and treatment. Medical doctors and dentists should in particular focus attention on those patients to anticipate clinical impairment.
